# Two phase II trials of temozolomide with interferon-*α*2b (pegylated and non-pegylated) in patients with recurrent glioblastoma multiforme

**DOI:** 10.1038/sj.bjc.6605189

**Published:** 2009-08-11

**Authors:** M D Groves, V K Puduvalli, M R Gilbert, V A Levin, C A Conrad, V H Liu, K Hunter, C Meyers, K R Hess, W K Alfred Yung

**Affiliations:** 1 Department of Neuro-Oncology, The University of Texas MD Anderson Cancer Center, Houston, TX, USA; 2 Department of Biostatistics, The University of Texas MD Anderson Cancer Center, Houston, TX, USA

**Keywords:** glioblastoma, multiforme, temozolomide, interferon, phase II

## Abstract

**Background:**

Because of the poor outcomes for patients with recurrent glioblastoma multiforme (GBM), and some laboratory and clinical evidence of efficacy using interferon in GBM, we assessed the toxicity and efficacy of temozolomide (TMZ) combined with either short-acting (IFN) or long-acting (pegylated) interferon *α*2b (PEG) in two single-arm phase II studies, and compared the results to 6-month progression-free survival (PFS-6) data from historical controls.

**Methods:**

Two single-arm phase II studies were carried out in adults with GBM. Patients were treated with the standard regimen of TMZ (150–200 mg m^−2^ per day × 5 days every month) combined with either 4 million units per m^2^ subcutaneously (SQ) three times weekly of IFN or 0.5 *μ*g kg^−1^ SQ weekly of PEG. Physical exams and imaging evaluations were carried out every 8 weeks.

**Results:**

On the IFN study, 34 adults (74% men) were enrolled, and 29 adults (55% men) on the PEG study; median Karnofsky performance status was 80 and 90 for the IFN and PEG studies, respectively. Grade 3 or 4 toxicities were common, leucopoenia and thrombocytopoenia occurring in 35–38% and 18–21% of patients, respectively. Grade 3 or 4 fatigue occurred in 18% of patients on both studies. Lymphopoenia was infrequent. PFS-6 was 31% for 29 evaluable patients in the IFN study and 38% for 26 evaluable patients in the PEG study.

**Conclusion:**

In recurrent GBM patients, both studies of standard dose TMZ with either IFN or PEG showed improved efficacy when compared to historical controls, or reports using TMZ alone. Even though the TMZ+PEG study met criteria for further study, the results of both of these studies must be considered in light of the standard of care (TMZ plus radiotherapy) for newly diagnosed GBM, which has evolved since the inception of these studies. Despite the results of the current studies being eclipsed by the new GBM standard of care, these results can still inform the development of newer approaches for GBM, either in an earlier, upfront setting, or by extrapolation of the results and consideration of the use of PEG or IFN in conjunction with other antiglioma strategies.

Nearly 9000 persons every year are diagnosed with glioblastoma (GBM) (CBTRUS, 2002) in the United States, and most of them die within 15 months of diagnosis due to tumour progression. Deaths occur despite aggressive surgical resection, followed by the current standard of involved-field radiation plus temozolomide (TMZ) followed by adjuvant postradiation TMZ chemotherapy (Stupp *et al*, 2005). Glioblastomas contain numerous genetic and epigenetic alterations that allow for their initiation and resistance to treatment, but a complete understanding of GBM biology is lacking (Ohgaki and Kleihues, 2007). Due to the incomplete understanding of GBM biology, difficulties with central nervous system (CNS) drug delivery, lack of availability of specific and targeted drugs and other logistic factors, no targeted or cytotoxic agent has surfaced as a clearly effective treatment for recurrent GBM patients.

Interferons (IFNs) are naturally occurring glycoproteins with immunomodulatory, antiproliferative, and antiangiogenic effects (Kirkwood and Ernstoff, 1984; Blanco *et al*, 2001; Jonasch and Haluska, 2001; Maher *et al*, 2007). The type I IFNs, *α* and *β*, are used in various malignant diseases (Maher *et al*, 2007). Numerous studies in glioma patients using both *α* and *β* IFNs have demonstrated possible efficacy. In recurrent GBM patients treated with intravenous IFN*β*, combined response rates (objective response and stable disease) of 51% (Yung *et al*, 1991) were reported. Other trials using IFN*β* have studied both recurrent GBM and postradiotherapy GBM patients who were stable (Fine *et al*, 1997; Colman *et al*, 2006) with suggestions of benefit. Varying degrees of efficacy have been demonstrated using IFN*α* in recurrent primary malignant gliomas, either alone or in combination with other modalities (Boethius *et al*, 1983; Hirakawa *et al*, 1983; Nakagawa *et al*, 1983; Nagai and Arai, 1984; Mahaley *et al*, 1985; Obbens *et al*, 1985; Hamada *et al*, 1986; Jereb *et al*, 1989; Yoshida *et al*, 1994; Buckner *et al*, 1995; Dillman *et al*, 1995; Brandes *et al*, 1997; Rajkumar *et al*, 1998). However, a large phase III trial compared postradiation BCNU either with or without IFN*α*, and did not show a benefit with the addition of IFN*α* (Buckner *et al*, 2001). A recent report of a small number of GBM patients suggested a possible inverse correlation between IFN*α* gene content and response to high-dose IFN*α* (Olson *et al*, 2004).

IFN*α*2b is a highly purified human protein produced by recombinant DNA techniques with indications for several malignant diseases. A formulation of IFN*α*2b using polyethylene glycol (PEG-Intron, peg IFN*α*2b) was developed to allow for longer patient exposure to drug. Conjugation of IFN*α*2b with polyethylene glycol significantly increases its half life (*t* 1/2) from approximately 4.0 to 40.3 hours, allowing for once a week (QW) dosing.

Temozolomide is a well-tolerated, orally bioavailable alkylating agent, approved by the Food and Drug Administration (FDA) for the adjuvant treatment of patients with GBM during and after radiotherapy. This regimen resulted in a median survival of 14.6 months and a 2-year survival of 26% (Stupp *et al*, 2005). Before its approval in the newly diagnosed GBM, TMZ was commonly used as a cytotoxic agent in combination with a variety of other antiglioma agents.

Because of the previous data suggesting activity of IFNs in recurrent GBM, we undertook the studies reported here to determine whether IFN*α*2b (standard and pegylated formulation) along with TMZ at the standard 5-day dosing scheme could improve 6-month progression-free survival (PFS-6) in patients with recurrent GBM. As all type I IFNs bind to the same two-subunit receptor, no significant difference in activity was expected between IFN*α* or -*β* (Maher *et al*, 2007). Interferon-*α*2b was chosen based on its broader history of use in malignant diseases, and on its ease of acquisition from the pharmaceutical sponsor of the studies. Results of two studies, TMZ plus IFN*α*2b (IFN) and TMZ plus pegylated IFN*α*2b (PEG) are presented, and both demonstrate benefit in prolonging PFS-6.

## Patients and methods

### Inclusion criteria

To be included in these studies, adult patients (⩾18 years old) with a Karnofsky performance status (KPS) of ⩾60% must have had intracranial GBM with evidence of tumour recurrence on CT or MRI scan of the brain. They must have failed previous radiation therapy (completed greater than 4 weeks before enrolment), and be on a stable dose of steroids. Patients may have had up to two previous chemotherapy regimens for not more than two previous relapses, and no exposure to either study drug. Patients were required to sign an institutional review board-approved informed consent, and to have recovered from effects of previous chemotherapy and have adequate organ function.

### Study design

#### Treatment, doses, and evaluations

Both studies were single-arm, open label phase II studies. Both studies used TMZ on a days 1–5 of the 28-day schedule at a starting dose of 200 mg m^−2^ for patients not previously treated with any chemotherapy or at 150 mg m^−2^ for patients who had received previous chemotherapy. The earlier study used standard interferon *α*2b (IFN) and the later study used PEG. Interferon was dosed at 4 million units (MU) m^−2^ per day subcutaneously (SQ) 3 days per week (Monday, Wednesday, Friday), days 8 through 28 of each 28-day course. PEG 0.5 *μ*g kg^−1^ was administered by SQ injection every week. Treatment courses were repeated every 28 days if all toxicities from the previous course have resolved to grade 2 or less. For continuation on study drugs, patients were required to have recovered haematologically to an absolute neutrophil count of ⩾1500 per *μ*l, platelet count ⩾100 000 per *μ*l, and all drug associated non-haematological toxicities had to have recovered to either grade 0 or 1. Drugs were held until recovery from toxicity had occurred. If recovery had not occurred by day 28, the subsequent course of TMZ was delayed until the toxicity criteria noted above were met. The dose of TMZ in subsequent courses was individually titrated and continued without interruption unless tumour recurrence or progression, and if toxicity was acceptable. Continuation of therapy after the first year was decided on an individual basis. For grade 3 or greater toxicities, treatment was withheld and the patients were monitored until toxicities resolved to grade 2 or better, then subsequent courses were started. A minimum of a 2-week rest period was required for grade 3 or greater toxicity. Dosages for subsequent courses were one dose level below the dose that produced toxicity of grade 3 or greater. Dose modifications were allowed based on patient tolerance. PEG was maintained at the starting dose if well tolerated, but dose deescalation by 50% (0.25 and then to 0.125 *μ*g kg^−1^ per week) was allowed. Interferon was started at 4 MU m^−2^ and could be increased to 5 or 6 MU m^−2^, or decreased to 3 or 2 MU m^−2^ as needed. Temozolomide could be deescalated to 175 or 150 mg m^−2^ (no previous TMZ exposure) or 125 or 100 mg m^−2^ (previous TMZ exposure) based on drug tolerance. Only two dose deescalations of IFN, PEG, or TMZ were permitted. Patients experiencing grade 3 or greater toxicities after two dose reductions were taken off the study. All patients were maintained on the lowest steroid dose necessary.

A minimum of 8 weeks of treatment was required for a patient to be considered as having received an adequate trial to evaluate efficacy. All patients were evaluable for toxicity and were followed for at least 30 days after completion of treatment. Any patient who progressed clinically during the first 8 weeks of therapy was evaluated by brain MRI scan and considered a protocol failure if disease progression was documented by neuroimaging. Patients who, for reasons other than clinical progression, discontinued treatment before the 8-week time point were not considered evaluable for efficacy of the treatment and not included in the PFS-6 determination.

Before enrolment, patients underwent a complete history and neurological examination, KPS determination, and documentation of evaluable disease. Pretreatment tests included CBC and standard blood tests verifying normal organ function, neuropsychological testing battery and brief fatigue inventory (only some patients), and contrast enhanced (Gd-DPTA) brain MRI scan. Baseline blood tests and MRI scans were performed within 14 days of registration.

During participation in the study, patients had routine blood testing (CBC and organ function tests) on day 22 of every 28-day course. Every 8 weeks, before odd-numbered courses, patients underwent contrast-enhanced brain MRI scans, physical and neurologic examinations with KPS and neuropsychological battery testing. A subset of patients also completed the brief fatigue inventory.

#### Outcome measures

The primary endpoint of the study was PFS-6, but patients were also observed for response to treatment, using a combination of the neurological examination and MRI brain scan to define overall response or progression. Bidimensional measurable lesions were necessary for response evaluation according to the guidelines promulgated by Macdonald *et al* (1990), and were required to be maintained for at least 4 weeks. Responses were only declared if patients were on no or stable maintenance doses of corticosteroids, had stable or improved neurological exams, and were determined, based on MRI changes, to have a reduction in tumour size. A complete response (CR) was complete disappearance of all measurable and evaluable disease and no new lesions, and no evidence of non-evaluable disease. A partial response (PR) was defined as ⩾50% decrease in the sum of products of perpendicular diameters of all measurable lesions, and no new lesions. Stable disease patients were those who did not qualify for CR, PR, or progression, and required a minimum of 12 weeks duration. Progression was declared when the MRI scan revealed a ⩾25% increase in the sum of products of all measurable lesions over the smallest sum observed (over baseline if no decrease), or appearance of any new lesion/site, or failure to return for evaluation due to death or deteriorating condition. Although the neurological examination was not used for determining response, it was noted and recorded and used in conjunction with the imaging data to determine a patient's overall clinical status. Patients were described as normal, improved from on-study state, the same as on-study state, or worse than on-study state (patient worse). Time to treatment failure was determined from the date of registration to the date of first observation of progressive disease, non-reversible neurologic progression, death due to any cause, or early discontinuation of treatment. Survival was determined from the date of registration to the date of death (due to any cause). Steroid dosage was carefully monitored and recorded during each course of therapy, and steroid dosage changes were considered before response determinations are made. NCI Clinical Toxicity Criteria, applicable to the time frame for each study, was used for toxicity determinations.

### Statistical analysis

The primary endpoint for both studies was improvement in PFS-6 (26 weeks). The historical values for comparison were from a database of 225 recurrent GBM patients enrolled in 8 previous phase II studies (in which none of the treatments were particularly effective; Wong *et al*, 1999). The proportion of patients remaining alive and free from progression at 6 months was 15% in that report.

Both studies were single arm by design. For the IFN study, hypotheses were H_0_: *P*⩽*P*0 *vs* H1: *P*⩾*P*1, where *P* was the probability of remaining alive and free from progression at 6 months; *α* (false positive rate) was=10% and *β* (false negative rate)=5%. *P*0 was set to 15% (95% CI for the proportion alive and FFP at 6 months was from 17 to 26%). *p*1 was set to 35%, looking for an improvement of 0.2. The plan was to declare success if >22.5% of patients were alive and free from progression at 6 months, with an *α* of 7% and a *β* of 6%. A 35% reponse (PFS-6) would yield a 95% CI on the true response proportion from 21 to 52%.

For the PEG study, a Simon's optimal two-stage design was employed. The hypotheses to be tested were H_0_: *p*⩽*p*0 *vs* H_1_: *p*⩾*p*1, where *p* is the probability of remaining alive and free from progression at 6 months; *α* was 10%, and *β* was 5%. *p*0 was set to 10% (The 95% confidence interval for our estimate of the proportion alive and progression free at 6 months was from 10 to 19%.) *p*1 was set to 30% (looking for an improvement of 0.2). These parameters led to a trial with a first stage of 20 patients (stopping if 2 or fewer patients respond) and a total of 40 patients (declaring success if more than 6 patients respond). As the outcome was observed over a 6-month period, it was necessary to suspend enrolment after the first 20 patients were enrolled to determine if the trial should be stopped or continued.

## Results

### Patients

For the earlier IFN study, 34 GBM patients were enrolled in 1998 in less than 5.5 months. For the later PEG study, 29 GBM patients were enrolled over 39 months from September 2002 through December 2005. In the PEG study, accrual was halted because of the establishment of the use of temozolomide as first line treatment, markedly reducing the number of potentially eligible patients. Median time from completion of radiation to enrolment was 24 weeks (range 4–456 weeks) for the IFN study and 22 weeks (range 4–324 weeks) for the PEG study. In the IFN study, 9 of 34 (26%) patients were enrolled within 3 months of completing radiation and for the PEG study, this figure was 7 of 29 (24%). Details of patient demographics are set forth in Table 1. There were differences between the two groups in the IFN and PEG studies, but using multivariable logistic regression analysis, no statistically significant differences were identified between the IFN and PEG groups with respect to age at diagnosis, KPS at enrolment, gender, previous extent of resection, or number of previous chemotherapies (all results: *P*>0.05).

### Response and survival

**Figure 1:**
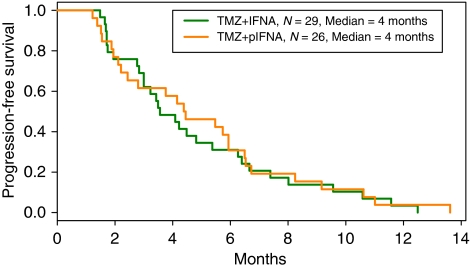
PFS-6 results for TMZ+IFN and TMZ+PEG.

Eight patients were not evaluable for response and these patients were not included in the survival analysis. One patient never received treatment (IFN study), two developed intercurrent illnesses that caused treatment discontinuance before treatment 1 course was administered (both in the PEG study) and five were lost to follow-up (four from the IFN study and one from the PEG study). Six-month progression-free survival was 31% for 29 evaluable patients in the IFN group and 38% for 26 evaluable patients in the PEG group. Kaplan–Meier survival curves for PFS-6 are shown in Figure 1. Results for patients enrolled within the first 3 months after completing radiation (patients with possible pseudoprogression) were a PFS-6 of 12.5% for the IFN study (*n*=9) and 57% for the PEG study (*n*=7). Excluding those patients enrolled within 3 months after completing radiation, leaves the IFN study with a PFS-6 of 40% and the PEG study with a PFS-6 of 35%. Additional response and PFS data are presented in Table 2. Using multivariate Cox proportional hazard regression analysis, the hazard ratio for progression for the PEG group was 0.63 (95% confidence interval: 0.3, 1.2, *P*=0.15). Due to wide a confidence interval, these results are inconclusive.

### Adverse events

All patients except the one from the IFN study who received no treatment are included in the report of toxicities. In the IFN and PEG groups, 20 of 33 (60%) and 21 of 29 (72%), respectively, of patients suffered at least one grade 3 or 4 toxicity event. Details of the grade 3 and 4 toxicities are presented in Table 3. Of those patients who had not received previous chemotherapy, 7 of 23 (30%) suffered grade 3 or greater thrombocytopoenia compared to 4 of 40 (10%) of those who had previously received chemotherapy. Grade 3 or greater leucopoenia occurred in 9 of 23 (39%) and 13 of 40 (33%) of patients who had no previous chemotherapy *vs* those who had received previous chemotherapy, respectively. Thus, previous exposure to chemotherapy did not appear to increase the risk of grade 3 or greater haematotoxicity in either study. However, this may have been due to lower TMZ dosing in those patients with previous chemotherapy exposure.

### Neurocognitive testing

Only 11 patients from the PEG study had baseline and at least 2 follow-up neurocognitive assessments. Very little cognitive decline was seen in these patients, but the quantity of data was felt insufficient from which to model statistically significant changes or make conclusions.

## Discussion

The current studies were conceived and their enrolment initiated before FDA approval of TMZ for GBM patients. The 2005 FDA approval of TMZ for GBM in the upfront setting, and the subsequent widespread adoption of this regimen (Stupp *et al*, 2005) resulted in the closure of the PEG study because of low availability of non-TMZ-treated patients and slow accrual. In the future, studies using modified dosing schedules of TMZ and/or combinations of TMZ with newer agents designed to overcome TMZ resistance might be necessary to ensure adequate accrual. Additionally, because of the use of TMZ with radiotherapy in the upfront setting in GBM, these results are somewhat anachronistic. But this fact does not diminish the study results when compared to the older data. The new data can serve as building blocks for future studies of interferons in different settings in glioma.

Despite the incomplete accrual of the second study, we believe these results provide interesting information, especially when compared to single-agent TMZ use and to the historical data. Based on the original statistical designs, the PEG study, but not the IFN study, met criteria as potentially worthy of further study. The PFS-6 results of 31 and 38% for IFN and PEG, respectively, compare favourably to the PFS-6 of 21% reported with standard (5-day every 28 days) dose TMZ in recurrent GBM (Yung *et al*, 2000). Even though the demographic differences (higher enrolment KPS, increased percentage of gross total resection, and lower number of previous chemotherapies) between the patients in the PEG *vs* IFN group did not meet statistical significance, these differences, favouring the PEG group, could have impacted outcomes.

The reasons for the improved outcomes reported here are not known, although some hypotheses can be offered. The short arm of chromosome 9, which contains the IFN gene cluster, is commonly lost in both primary and secondary GBMs (Maher *et al*, 2006), as well as in recurrent high-grade tumours when compared to low-grade tumours (Kim *et al*, 1995; Weber *et al*, 1996). There is evidence that the short arm of chromosome 9 may contains genes (IFN or others) with tumour suppressor activity (James *et al*, 1991, 1993). Malignant glioma specimens are reported to have a 50% rate of deletion of the IFN*α* gene cluster (James *et al*, 1991). Further, IFN*α* and -*β* gene deletions are reported in 26–46% of glioma cell lines (Miyakoshi *et al*, 1990; James *et al*, 1993). These data notwithstanding, *in vitro* experiments suggest an antineoplastic action of IFN treatment, regardless of the IFN gene status of tumour cells (Miyakoshi *et al*, 1990). Replacement of IFN protein may, through a variety of mechanisms, result in an antitumour effect. Interferon-*α* results in both direct and indirect immune-mediated effects (Barth and Morton, 1995), which may be relevant to the effects seen here. Antiproliferative, antiangiogenic, and prodifferentiation effects may also be contributing to the effects seen. Some evidence also suggests IFNs may downregulate MGMT, increasing tumour cell sensitivity to TMZ (Natsume *et al*, 2005).

A few features of the adverse event profile of these drug combinations warrant discussion. First, the risk of grade 3 or 4 anaemia and leucopoenia are almost equivalent to single-agent TMZ (temozolomide package insert). However, the risk of lymphopoenia (3%) was remarkably lower than that reported for single-agent TMZ (55%). Further, no pneumocystis pneumonia was observed in these patients. Additionally, the risk of thrombotic events was higher in the PEG-treated patients (17%), as compared to the IFN-treated ones (0%). The reasons for these observations are not clear from the literature as well as after review of the specific patient histories.

Even though the studies reported here appear to show some benefit over standard dose TMZ in recurrent GBM, several factors may militate against these results. As both studies are single institution and non-randomised, their results are less reliable and may reflect a referral bias. In addition, pseudoprogression at the time of enrolment in some patients cannot be completely excluded as a possible contributor to the improved outcomes, despite its apparent lack of impact when one excludes from analysis those patients enrolled within 3 months after the completion of radiotherapy.

## Conclusions

The two phase II studies demonstrated some improvement in PFS-6 outcomes in recurrent GBM patients as compared with historical data and with the single-agent TMZ data. The PEG study met criteria to be deemed worthy of further investigation. The use of this combination and dosing scheme in the recurrent setting is probably not feasible at this time, due to the near universal exposure of GBM patients to TMZ as an up-front treatment. Possible future avenues of investigation of PEG in malignant glioma patients could include (1) PEG combined with TMZ and XRT in the up-front GBM setting (using MGMT methylation status or IFN*α* gene content as an enrolment criteria), (2) the addition of IFN or PEG to an intensified TMZ regimen at recurrence (Wick *et al*, 2007), (3) the addition of PEG to alternative cytotoxic chemotherapies, looking for improvement in outcomes, or (4) the addition of PEG to other non-cytotoxic therapies, as a potential synergistic agent.

## Figures and Tables

**Table 1:** Patient demographics

**Parameter**	**TMZ+IFN**	**TMZ+PEG**
Number of patients	34	29
Age: median (range)	55 (17–69)	56 (20–67)
Gender: M/F (%)	25 : 9 (74:26)	16 : 13 (55 : 45)
		
*KPS*		
Median	80	90
100%	3 (9%)	5 (17%)
90%	7 (21%)	16 (55%)
80%	17 (50%)	5 (17%)
70%	7 (21%)	3 (10%)
		
*Maximum prior resection*		
Biopsy	2 (6%)	5 (17%)
Subtotal resection	20 (59%)	9 (31%)
Gross total resection	12 (35%)	15 (52%)
		
*Number of prior chemotherapies*		
0	9 (26%)	14 (48%)
1	24 (71%)^a^	14 (48%)^b^
2	1 (3%)	1 (3%)

a Prior chemotherapies included: topotecan, BCNU, cisplatinum, procarbazine+CCNU+vincristine (PCV), hydroxyurea, marimastat, 6-thioguanine, 13-cis-retinoic acid, carboplatin, and etoposide.

b Prior chemotherapies included: celecoxib, gefitinib, BCNU, CCNU, vincristine, 13-cis-retinoic acid, PCV, tipifarnib, and erlotinib.

**Table 2:** Response data

**Study**	**6-Month PFS % (95% CI)**	**Median PFS months (95% CI)**	**Median OS months (95% CI)**	**% Responders (all partial)**	**% Stable disease**	**% Responders+stable disease**
TMZ+IFN; *n*=34	31 (16–54)^a^	3.6 (3.0–6.3)	7.2 (5.3–10.6)	4/34 (12%)	18/34 (53%)	22/34 (65%)
TMZ+PEG; *n*=29	38 (24–64)^a^	4.4 (2.4–6.5)	10.0 (7.8–14.3)	1/29 (3%)	17/29 (57%)	18/29 (62%)

a Numbers are *n*=29 for TMZ+IFN and *n*=26 for TMZ+PEG due to patients being not evaluable for response.

**Table 3:** Grade 3 and 4 adverse events

**Grade 3 or 4 toxicity**	**IFN study (*N*=33): *n* (%)**	**PEG study (*N*=29): *n* (%)**
Leucopoenia	12 (35)	11 (38)
Thrombocytopoenia	6 (18)	6 (21)
Fatigue	6 (18)	6 (18)
Pulmonary embolus/thrombosis	0 (0)	5 (17)
Nausea/vomiting	2 (6)	0 (0)
Headache	0 (0)	2 (7)
Incontinence	0 (0)	2 (7)
Other	2 (3)^a^	8 (3)^b^

a One instance each of diarrhoea and elevated transaminases.

b One instance each of anaemia, confusion, depression, hypotension, hypoxemia, infection, lymphopoenia, and pruritis.
